# Supplementary material for: Time‑resolved multi-omic analysis of paclitaxel exposure in human iPSC‑derived sensory neurons unveils mechanisms of chemotherapy‑induced peripheral neuropathy
Source: Cell Death Dis. 2026 Feb 10;17(1):211. doi: 10.1038/s41419-026-08445-2 (PMC12921266; doi:10.1038/s41419-026-08445-2)
Supplement: Supplementary file 15 — Dataset 1. [file 41419_2026_8445_MOESM15_ESM.zip › CDD_Open_Data/04_RNA_Seq/Timeline_panel_plots_etc_2023-01-23_1609.zip!/Panel_Plots_etc/Evidence_Plots/post5d/evidence_plot_standard contrasts_msigdb_reactome_5d_PTX_vs_DMSO_ID6_M27617_REACTOME_PROTEIN_PROTEIN_INTERACTIONS_AT_SYNAPSES.pdf]

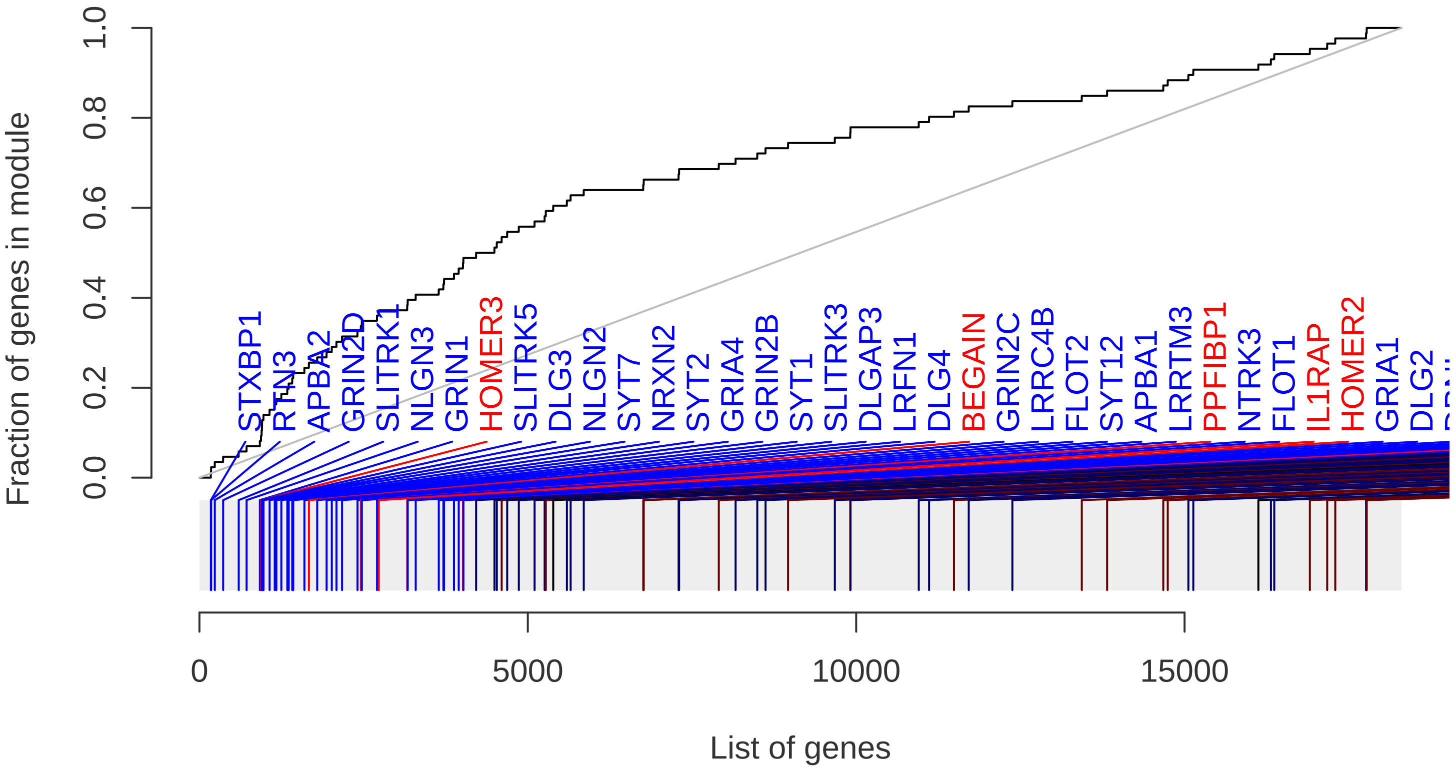

Fraction of genes in module
0.0
0.2
0.4
0.6
0.8
1.0
STXBP1
RTN3
APBA2
GRIN2D
SLITRK1
NLGN3
GRIN1
HOMER3
SLITRK5
DLG3
NLGN2
SYT7
NRXN2
SYT2
GRIA4
GRIN2B
SYT1
SLITRK3
DLGAP3
LRFN1
DLG4
BEGAIN
GRIN2C
LRRC4B
FLOT2
SYT12
APBA1
LRRTM3
PPFIBP1
NTRK3
FLOT1
IL1RAP
HOMER2
GRIA1
DLG2
0
5000
10000
15000
List of genes
